# Therapeutic Potential of Iron Chelating Therapy: Modification of Disease by Iron Depletion

**DOI:** 10.1155/MBD.1994.83

**Published:** 1994

**Authors:** Chaim Hershko, Gabriela Link, Miryam Tzahor, Arie Pinson

**Affiliations:** 1 Department of Medicine Shaare Zedek Medical Center Jerusalem 91031 Israel; 2 Department of Human Nutrition and Metabolism Hebrew University Hadassah Medical School Jerusalem 91031 Israel; 3 Laboratory for Myocardial Research Hebrew University Hadassah Medical School Jerusalem 91031 Israel


### THERAPEUTIC POTENTIAL OF IRON CHELATING THERAPY: MODIFICATION OF DISEASE BY IRON DEPLETION

Chaim Hershko*[1], Gabriela Link[2], Miryam Tzahor[2] and Arie Pinson[3]

[1] Department of Medicine, Shaare Zedek Medical Center, Jerusalem 91031, Israel
[2] Department of Human Nutrition and Metabolism and
[3] Laboratory for Myocardial Research,
Hebrew University Hadassah Medical School, Jerusalem 91031, Israel

After a delay of almost two decades, desferrioxamine (DF) has been reintroduced for clinical use by the pioneering work of British investigators such as Michael Berry, Bernadette Modell, Martin Pippard and Victor Hoffbrand (1). The widespread use of DF was firmly established thanks to the exceptional devotion of our Italian colleagues. In the following, I shall focus on the impact of iron chelating treatment on survival in Thalassemia; examine the mechanism whereby DF protects the heart from iron toxicity, and: examine the potential usefulness of iron chelators in conditions unrelated to iron overload.

(I) Management of transfusional iron overload: Survival in thalassemic patients in recent years is definitely better than in the pre-DF era. The most extensive and detailed information on survival in thalassemia comes from Italy, where over 5000 patients with thalassemia major are treated and monitored by a remarkably well coordinated national study group of the National Association for Pediatric Oncology and Hematology (AIEOP). In Italy continuous subcutaneous DF treatment has been introduced and used systematically since 1978 (2). In a report by Borgna-Pignatti et al (3) on 942 thalassemic patients from Italy, a steady improvement in survival has been shown for cohorts born in 1960-1964, 1965-1969 and 1970-1974. The total mortality was 15.7% and heart disease was the cause of death in 2/3 of cases. Overall survival from birth for patients born in 1970-1974 and therefore subjected to chelation therapy from an early age, was 97% at 10 years and 94% at 15 years. These impressive figures illustrate the significant improvement that has taken place in recent years in the survival of patients with thalassemia major receiving adequate therapy.

(II) Effect of iron toxicity and chelation on cultured heart cells: Because the most important target of iron toxicity is the heart, we have developed an experimental model of rat heart cell cultures in which the harmful effects of iron overload and the beneficial effects of iron chelating treatment may be studied with complete control of the extracellular environment (4). A unique feature of this model is the ability to correlate functional abnormalities in heart cell contractility and rhythmicity induced by iron toxicity, with structural aberrations such as alterations in membrane lipid and protein composition. Our studies in cultured heart cells have shown that 24-hour incubation with iron at concentrations ranging from 20 to 80 micrograms per ml resulted in a drastic decrease in the amplitude and rate of contractions, a dose-related decrease in the magnitude of the calcium-dependent overshoot potential, and gross abnormalities in rhythmicity resembling ventricular fibrillation in 20 to 30 % of culture plates (5). All of these abnormalities were abolished by subsequent treatment with DF at a concentration of 0.3 mM.

Despite enhanced lipid peroxidation indicated by increased malondialdehyde production (6), alterations in sarcolemmal lipid composition were negligible. In contrast, there was a marked decrease in the activity of the thiolic sarcolemmal enzymes 5'-nucleotidase and $Na^+K^+$-ATPase, reversed completely by DF treatment. Conversely, iron-loading resulted in a sharp increase in the total activity, increased free activity and loss of latent activity of the lysosomal enzyme ß-hexosaminidase, implying increased lysosomal fragility (7). These observations indicate that, in addition to increased lysosomal fragility, damage to sarcolemmal thiolic enzymes is an important primary effect of myocardial iron toxicity. Since $Na^+K^+$-ATPase is directly involved in membrane ion transport and $Ca^{++}$ regulation, damage to thiolic sarcolemmal enzymes may be responsible for the abnormalities in myocardial function associated with iron overload.

(III) Reperfusion injury: When vital organs are subjected to hypoxia and then exposed to oxygen during reperfusion, harmful oxygen species are formed which may aggravate the damage caused by anoxia. This additional damage is referred to as "reperfusion injury". Superoxide and peroxide are poorly reactive in aqueous solution, but the hydroxyl radical is a highly reactive, toxic species. Hydroxyl radicals are produced through the Haber-Weiss reaction which is catalyzed by iron. Conversely, DF is very efficient in preventing the participation of iron in the Haber-Weiss reaction and therefore may prevent reperfusion injury. Prevention of reperfusion injury may have important implications to post-ischemic damage to the brain or heart, and to the preservation of organs for transplantation (8,9).

Because of the importance of introducing the chelator prior to reperfusion damage, the most promising situations in clinical medicine where DF may be useful are elective surgical procedures and organ transplantation wherein DF treatment may be started before and throughout cardioplegia and hypothermic anoxia.

(IV) Cell proliferation: The proliferation of mammalian cells is inhibited by severe iron deficiency. A similar inhibition of DNA synthesis may be achieved by in vitro DF treatment of human lymphocytes (10). The mechanism of this inhibition by DF is interaction with ribonucleotide reductase, a rate-controlling enzyme in DNA synthesis. This enzyme contains a tyrosine free-radical structure which is essential for its activity, and which requires the presence of iron and oxygen . The binding of chelatable iron by DF results in the inactivation of ribonucleotide reductase, similar to the effect of hydroxyurea, a known inhibitor of this enzyme . Important potential applications of iron chelators in this respect are inhibition of tumour cell growth such as in neuroblastoma and other malignancies (11), and inhibition of parasite growth in malaria (12,13) and other parasitic infections.

(V) Drug toxicity: Iron plays an important role in the formation of oxygen radicals by anthracyclines. Daunorubicin or doxorubicin are reduced to a semiquinone free radical which, in turn, reduces molecular oxygen to the superoxide ion. In studies performed recently in our laboratory, (14) we wished to test the hypothesis that iron may have a role in anthracycline cardiotoxicity and, conversely, that such toxicity may be inhibited by iron chelators. These studies have shown that iron-loading prior to doxorubicin treatment causes a 2 to 3-fold increase in doxorubicin toxicity manifested in LDH release, a sharp decrease in spontaneous heart cell beating, and the emergence of irregular and ineffective heart cell beats. DF treatment of iron-loaded cells prior to doxorubicin therapy resulted in decreased doxorubicin toxicity as manifested in all of the above abnormalities. These observations provide strong support for the claim that iron- loading may aggravate and iron chelating treatment may limit the cardiotoxicity of anthracycline drugs.

Conclusions: There can be no doubt today that DF has changed the quality of life and life expectancy of many patients with thalassemia. The important new observations on the modification of disease by preventing the formation of free radicals, and the demonstration of the antiproliferative and antimicrobial effects of iron chelators lend new meaning to the term "Nutritional Immunity" and open new channels for exploring the possibility of controlling disease by selective intracellular iron deprivation. Packaging the chelators in liposomes or red cell ghosts, or manipulating their lipid solubility to improve their delivery to appropriate target organs such as the macrophage system may greatly improve their efficiency. In view of the short half-life of DF and its poor oral effectiveness it is unlikely that DF per se will be suitable for clinical use as a practical antimicrobial or antiproliferative agent. However, with the introduction of simple, orally effective new chelators, it is reasonable to expect that future research may lead to the identification of iron chelators with considerable usefulness in the control of a wide range of clinical problems.

### REFERENCES

1. Hershko, C., and Weatherall, D.J., Iron chelating therapy. CRC Crit. Rev. Clin. Lab. Sci. 26, 303, 1988.

2. Gabutti, V., Current therapy for thalassemia in Italy, in A Bank Ed. Sixth Cooley's Anemia Symposium, Ann. New York Acad. Sci 612, 268, 1990.

3. Borgna-Pignatti, C., Zurlo, M.G., DeStefano, P., DiGregorio, F., Di Palma, A., Piga, A., Melevendi, C., Burattini, M.G., Terzoli, S., and Masera G., Survival in thalassemia with conventional treatment. in Buckner C D, Gale R P, and Lucarelli G eds. Advances and Controversies in Thalassemia Therapy: Bone Marrow Transplantation and Other Approaches. Alan R Liss Inc. New York. 27, 1989.

4. Link, G., Pinson, A. and Hershko, C., Heart cells in culture: a model of myocardial iron overload and chelation, J. Lab. Clin. Med., 106, 147, 1985.

5. Link, G., Athias, P., Grynberg, A., Pinson, A. and Hershko, C., Effect of iron loading on transmembrane potential, contraction and automaticity of rat ventricular muscle cells in culture, J. Lab. Clin. Med., 113, 103, 1989.

6. Link, G., Pinson, A., Kahane, I. and Hershko, C., Iron loading modifies the fatty acid composition of cultured rat myocardial cells and liposomal vesicles: Effect of ascorbate and $\alpha$-tocopherol on myocardial lipid peroxidation, J Lab. Clin. Med., 114, 243, 1989.

7. Link G, Pinson A, Hershko C., Iron-loading of cultured cardiac myocytes modifies sarcolemmal structure and increases lysosomal fragility, J Lab Clin Med, in press, 1992.

8. Lesnefsky, E.J., Repine, J.E. and Horwitz, L.D., Deferoxamine pretreatment reduces canine infarct size and oxidative injury, J. Pharm. Exper. Therap., 253, 1103, 1990.

9. Menasche, P., Antebi, H., Alcindor, L.G., Teiger, E., Perez, G., Giudicelli, Y., Nordmann, R. and Piwnica, A., Iron chelation by deferoxamine inhibits lipid peroxidation during cardiopulmonary bypass in humans, Circulation, 82, (5 Suppl) IV-390, 1990.

10. Lederman, H.M., Cohen, A., Lee, J.W.W., Freedman, M.H. and Gelfand, E.W., Deferoxamine: a reversible S-phase inhibitor of human lymphocyte proliferation, Blood, 64, 748, 1984.

11. Donfrancesco, A., Deb, G., Dominici, C., Pileggi, D., Castello, M.A. and Helson, L., Effects of single course of deferoxamine in neuroblastoma patients, Cancer Res., 50, 4929, 1990.

12. Hershko, C.and Peto, T.E.A., Deferoxamine inhibition of malaria is independent of host iron status, J. Exper. Med., 168, 375, 1988.

13. Gordeuk, V.R., Thuma, P.E., Brittenham, G.M., Zulu, S., Simwanza, G., Mhangu, A., Flesch, G. and Parry, D., Iron chelation with deferoxamine B in adults with asymptomatic Plasmodium falciparum parasitemia. Blood, 79, 308, 1992.

14. Hershko, C., Link, G. and Pinson, A., Preventing anthracycline toxicity: Cardioprotective effect of iron chelating therapy in cultured rat heart cells. 3rd NIH Symposium on Development of Iron Chelators for Clinical Use. May 20-22, Gaineville, Florida. 1992, (abstract).

**Received: March 20, 1993**

